# Characterization of Thermo-Physical Properties of EVA/ATH: Application to Gasification Experiments and Pyrolysis Modeling

**DOI:** 10.3390/ma8115428

**Published:** 2015-11-20

**Authors:** Bertrand Girardin, Gaëlle Fontaine, Sophie Duquesne, Michael Försth, Serge Bourbigot

**Affiliations:** 1 Unité Matériaux et Transformations (UMET)-CNRS UMR 8207-Group Reaction and Resistance to Fire (R2Fire), École Nationale Supérieure de Chimie de Lille, University of Lille, Avenue Mendeleiev, CS 90108, 59652 Villeneuve d'Ascq Cedex, France; bgirardi@enscl.fr (B.G.); gaelle.fontaine@ensc-lille.fr (G.F.); sophie.duquesne@ensc-lille.fr (S.D.); 2 SP Fire Research, SP Technical Research Institute of Sweden, P.O. Box 857, SE-501 15 Borås, Sweden; Michael.Forsth@sp.se; 3 Department of Civil, Environmental and Natural Resources Engineering, Luleå University of Technology, SE-971 87 Luleå, Sweden

**Keywords:** pyrolysis modelling, thermal conductivity, heat capacity, gasification, ethylene vinyl acetate copolymer, aluminum tri-hydroxide

## Abstract

The pyrolysis of solid polymeric materials is a complex process that involves both chemical and physical phenomena such as phase transitions, chemical reactions, heat transfer, and mass transport of gaseous components. For modeling purposes, it is important to characterize and to quantify the properties driving those phenomena, especially in the case of flame-retarded materials. In this study, protocols have been developed to characterize the thermal conductivity and the heat capacity of an ethylene-vinyl acetate copolymer (EVA) flame retarded with aluminum tri-hydroxide (ATH). These properties were measured for the various species identified across the decomposition of the material. Namely, the thermal conductivity was found to decrease as a function of temperature before decomposition whereas the ceramic residue obtained after the decomposition at the steady state exhibits a thermal conductivity as low as 0.2 W/m/K. The heat capacity of the material was also investigated using both isothermal modulated Differential Scanning Calorimetry (DSC) and the standard method (ASTM E1269). It was shown that the final residue exhibits a similar behavior to alumina, which is consistent with the decomposition pathway of EVA/ATH. Besides, the two experimental approaches give similar results over the whole range of temperatures. Moreover, the optical properties before decomposition and the heat capacity of the decomposition gases were also analyzed. Those properties were then used as input data for a pyrolysis model in order to predict gasification experiments. Mass losses of gasification experiments were well predicted, thus validating the characterization of the thermo-physical properties of the material.

## 1. Introduction

One of the primary aspects of fire safety engineering is to model and/or to predict how a material or a structure will behave under a given fire scenario. A large variety of models for the condensed phase action, including mass and heat transport, have been developed during the last decade (Fire Dynamics Simulator pyrolysis sub-model [1], Gpyro [2], ThermaKin [3], or Pyropolis [4], for example). Despite those developments, pyrolysis models are seldom applied to complex objects or formulated materials. Indeed, to represent how a material behaves under a thermal constraint, several phenomena have to be taken into account which are not necessarily applicable from one type of material to another. As an example, delamination can occur for composites or fiber-reinforced materials, but is quite uncommon for pristine or filled polymers which involve dripping and/or charring [5].

At this date, there are different ways to numerically describe the ignition of a material. Namely, ignition can be described with a critical surface temperature (as it can be done in FDS) which is generally determined by conducting cone calorimeter experiments at different heat fluxes as proposed by Janssens [6,7]. Another solution is to use a critical mass flux for ignition (as proposed in ThermaKin) [3,8,9,10]. It describes the time when enough combustible gases are evolved from a degrading material and mixed with an oxidizer (generally the oxygen from the surrounding air) to produce a flame. However, the modeling of ignition is not an easy task, and thus the validation of the inputs used for pyrolysis models has to be done by the modeling of gasification experiments. Those experiments are generally performed using the Flame Propagation Apparatus or the controlled-atmosphere cone calorimeter as developed by Babrauskas *et al.* [11]. Since no ignition occurs as the materials undergo a pyrolytic decomposition and no oxidizer can ignite the decomposition products, there is no need for numerical parameters to describe ignition. This approach was, for example, used by Chaos *et al.* [12] and by Li *et al.* [13] to study the gasification of charring and non-charring polymers.

Pyrolysis models can be extremely complex and can potentially require a large amount of input parameters describing the material, namely its thermo-physical properties such as heat capacity, thermal conductivity, or density. Those inputs can be obtained by inverse methods and/or by direct measurements. In the first case, it can be argued that those parameters are not linked to real physical properties of the studied material as they are determined by optimization processes to obtain the best fit between experimental and simulated results. On the other hand, depending on the algorithm, the property to be optimized, and the experimental data, great discrepancies in the results can be obtained. As an example, Chaos *et al.* [12] found a heat capacity of 1.5 J/g/K and 2.5 J/g/K when simulating the same data but with different algorithms. This approach does not allow the estimation of reliable physical properties, and direct measurements are then preferable

The direct measurements of the above properties can be done using a thermo-gravimetric or calorimetric apparatus. Even if the characterization of thermal properties such as heat capacity, thermal conductivity, or thermal diffusivity is standardized [14,15,16,17,18], the main challenge for accurate pyrolysis modeling is to be able to measure those properties before, during, and after the decomposition of the material and also at high temperatures. This can be done by following these standards in the case of virgin materials [19,20,21]. On the contrary, it can be more challenging to measure the properties of the obtained residues at the temperature at which they are formed or at higher temperatures. Similarly, multiple-step decomposition materials are also difficult to characterize as the intermediate species have to be characterized even if their domain of stability are limited. Unfortunately, flame-retarded materials are generally formulated with multiple additives to improve thermal, mechanical, electrical, or aging properties, and thus they are materials that follow multiple steps reactions during their decomposition, and hence, it is difficult to apply numerical models. It is nonetheless possible and some studies can be found in the literature [22,23], but in those cases the fillers are assumed to be inert, which considerably simplifies the model.

Moreover, it can also be noted that the thermal properties should be expressed as a function of temperature, as their values can greatly change when the temperature of the material increases as it is exposed to an external heat flux. As an example, the heat capacity of alumina can vary from 0.78 J/g/K at 25 °C up to 1.24 J/g/K at 727 °C [24,25], corresponding to a 59% increase. Those variations of the thermal properties have to then be taken into account in the numerical models either by using temperature-dependent values or by using temperature-averaged values. This latter is easier to implement in the models and can be more easily obtained but is also less representative of the reality.

The aim of this study is to develop various characterization methods to determine thermo-physical properties of flame-retarded materials and to evaluate the influence of these methods on the pyrolysis model. In order to take into account the physical and chemical interactions between a flame retardant and a polymer, aluminum tri-hydroxide (ATH) and an ethylene-vinyl acetate copolymer (EVA) were selected. Indeed, industrial products such as low-smoke halogen-free cables are often based on polyolefin and ATH is widely used in industrial application for its flame-retardant properties. Such cables generally require a large amount of mineral filler (between 60 and 70 wt % in the case of ATH) [26,27] and synergists such as organo-clays [28,29]. In order to work with a material representative of industrial products, an EVA containing 65 wt % of ATH as a flame retardant was chosen. This material was fully characterized with a focus on the thermal properties as a function of the temperature. Afterward, the thermal decomposition of EVA/ATH formulation in a controlled atmosphere mass loss cone in anaerobic atmosphere, to avoid combustion of the sample, was investigated. Finally, the simulation of these gasification experiments allowed us to validate our experimental approach for the characterization of the input data.

## 2. Experimental Section 

### 2.1. Material and Sample Preparation

The polymer used in this study is an EVATANE^®^ 28-05 (hereafter called EVA), corresponding to an ethylene-vinyl acetate copolymer containing 28 wt % of vinyl acetate (MFI = 5–8 g/10 min). Aluminum trihydroxide (hereafter called ATH) was supplied in powder form from Nabaltec as Apyral^®^ 40CD (BET = 3.5 m²/g, D_50_ = 1.3 µm). The loading of ATH was fixed at 65 wt %.

**Figure 1:**
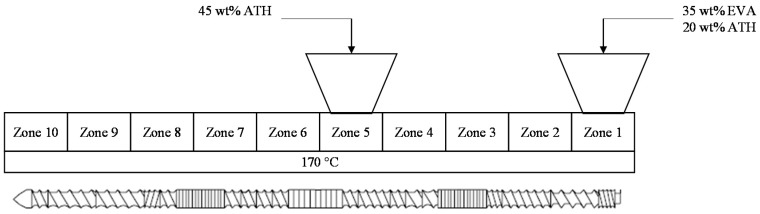
Extrusion process design with feed zones and temperature profile.

The samples were prepared with a co-rotating twin screw extruder from Thermo Fisher Scientific (HAAKE Rheomix OS PTW 16 twin screw extruder) with a barrel length of 400 mm and a screw diameter of 16 mm (L/D = 25). The extruder is composed of a heated barrel divided in 10 zones, in which the polymer melts. A flat temperature profile was used and all the zones were set at 170 °C. The rotational speed of the screws was set at 100 rpm. The EVA and 20 wt % of ATH were fed in zone 1 and the remaining 45 wt % of ATH were delivered in zone 5 as shown in Figure 1. This was done using volumetric feed-dosing elements. This design was used in order to avoid too-high torque during extrusion and to promote ATH dispersion in the EVA matrix. The obtained formulation (hereafter called EVA/ATH) was then pelletized.

A high speed rotor mill (Retsch-Ultra Centrifugal Mill ZM200) was used to obtain fine powder from the formulation pellets. Before grinding, the material was dipped in liquid nitrogen. The fed material only remained in the grinding chamber for a very short time, and thus, the features of the sample were not modified.

Specimens for the fire testing were obtained by pressing the appropriate mass of material, previously extruded and pelletized, with a hydraulic hot press using specific molds at 170 °C and under 40 kN for 10 min.

### 2.2. Thermal Conductivity

Thermal conductivity measurements were carried out, from 20 °C to 700 °C, using a Hot Disk thermal constant analyzer (TPS2500S) from Thermoconcept (Merignac, France), which is based on a Transient Plane Source method. The sensor, which acts both as a heater and a thermocouple, is hot pressed between two 25 × 25 × 7 mm^3^ plates of the material to ensure a good contact between the sensor and the sample. For each temperature, the measurements were performed as soon as the sample temperature was down to ±0.5 °C of the desired temperature for 30 min by applying a power of 0.02–0.09 W for 10–80 s, depending on the thermal properties of the sample. The furnace was directly connected to a nitrogen flow and all experiments were carried out in an inert atmosphere to prevent oxidation of the sensor. The experiments were run as duplicates and were repeatable within 5% margin of error.

### 2.3. Thermo-Gravimetric Analysis and Kinetic Analysis

Thermo-gravimetric analysis was carried out on a TA Instruments TGA Q5000IR. The balance purge flow was set at 15 mL/min and the sample purge flow (nitrogen) at 25 mL/min. Samples of exactly 10 mg (±0.3 mg) were put in open alumina pans (250 µL) and underwent heating from 50 to 800 °C with heating rates of 2, 10, 20, and 50 K/min. We verified for each heating rate that the buoyancy force was negligible, subtracting the thermo-gravimetric curve of the blank.

Kinetic analysis and modeling of the decomposition of the samples was made using an advanced Thermokinetic software package developed by Netzsch Company. The principle has been discussed in detail by Opfermann [30,31]. An example of application of this technique with EVA/ATH can be found elsewhere [32].

### 2.4. Differential Scanning Calorimetry

Differential Scanning Calorimetry experiments were conducted with a Netzsch 449 F1 Jupiter simultaneous thermal analyzer (STA). Samples of exactly 25 mg (±0.3 mg) were put in platinum/rhodium pans with lids. The lids had a small (0.25 mm in diameter) orifice for ventilation. This container configuration was used to maximize the thermal contact between a degrading sample and heat flow-sensing thermocouple located underneath the pan. We verified for each experiment that the buoyancy force was negligible, subtracting the TG curve of the blank. Temperature and sensitivity calibrations were conducted with the STA prior to testing the samples at a heating rate of 20 K/min. The temperature calibration relates the literature value of the melting temperatures of organic and inorganic samples to the melting temperature of the same materials measured by the apparatus. The sensitivity calibration relates the literature value of the enthalpy of the melting of organic and inorganic samples to the measured enthalpy of the melting of the same samples.

The heat capacity of the material was determined using two different techniques. First was isothermal modulated Differential Scanning Calorimetry (mDSC). The measurements were made at 50 °C and then every 100 °C from 100 °C to 800 °C with 20 K/min ramps between each isothermal step. At each temperature, an isothermal step of 120 min was used prior to the mDSC measurement using a modulation of ±0.5 K every 60 s. Secondly, heat capacity was determined according to ASTM E1269-11 [16]. Synthetic sapphire of known heat capacity [14] of 56.1 and 27.8 mg provided by Netzsch have been used as reference material. Heat capacity of the material is then given by Equation (1) as defined in the standard [14].

(1) $${\text{Cp}}_{\text{material}}=\frac{{\text{m}}_{\text{sapphire}}\times {\text{Heat Flow}}_{\text{material}}}{{\text{m}}_{\text{material}}\times {\text{Heat Flow}}_{\text{sapphire}}}\times {\text{Cp}}_{\text{sapphire}}$$

where *m*_sapphire_ and *m*_material_ are the instantaneous mass measured by the STA apparatus, Heat Flow_material_ and Heat Flow_sapphire_ are the intensity of the heat flow signal measured by the STA apparatus, corrected by a blank experiment taken into account for the crucible, and *Cp*_sapphire_ is extracted from the literature and given by the standard [14].

### 2.5. Optical Measurements

Transmittance and reflectance spectra in the UltraViolet, visible, and near Infra-Red region, with wavelengths in the interval 0.3–2.5 μm, were recorded using a Perkin Elmer Lambda 900 double-beam spectrophotometer equipped with an integrating sphere. In the infrared wavelength range (2.5–17 μm), a Brüker FTIR single-beam spectrophotometer equipped with a gold-coated integrating sphere was used. More details about the optical experimental setup are available in a recent paper published by Witkowski *et al.* [19].

The radiative properties of importance for modeling are the scalar surface absorptivity (α), and the scalar in-depth absorption coefficient (A). It is important to notice that those values have to be expressed independently of the incident wavelength λ if the modeling software is not able to handle spectrally resolved data. To be able to calculate the scalar absorptivity for incident radiation for a given irradiation spectrum, information is required on the spectrally resolved absorptivity α_λ_. The ratio between the total absorbed radiation and total incident radiation is given by the scalar absorptivity (α), as defined in Equation (2). This is the weighted averaged of the spectrally resolved absorptivity which is actually measured, where the weight function is the excitance spectrum of the radiation source (*M*_λ_), which is the heated coil of the cone calorimeter in this study.

It was shown by Boulet *et al.* [33] that *M*_λ_ is very well described by a black body spectrum. In this study, the irradiation level in the cone calorimeter was 50 kW/m², which corresponds to a coil temperature of 1025 K [34]. The excitance spectrum for our experiments therefore corresponds to a black body spectrum given by Planck’s law for 1025 K.

(2) $$\alpha =\frac{\int_{0}^{\infty }\alpha_{\lambda }M_{\lambda }d\lambda }{\int_{0}^{\infty }M_{\lambda }d\lambda }$$

The calculation and experiments for measuring the scalar in-depth absorption coefficient A are more complex. The spectrally resolved irradiance at a depth *x* into the sample is given by the Beer-Lambert law as defined in Equation (3). By integrating the contributions for all wavelengths, it is possible to evaluate the total irradiance at depth *x* as defined in Equation (4).

(3) $${\text{I}}_{\text{λ}}\left(\text{x}\right)={\text{I}}_{\text{λ}}\left(0\right){\text{e}}^{-{\text{A}}_{\text{λ}}\cdot \text{x}}$$

(4) $$\text{I}\left(\text{x}\right)=\int^{\text{​}}{\text{I}}_{\text{λ}}\left(0\right){\text{e}}^{-{\text{A}}_{\text{λ}}\cdot \text{x}}\text{dλ}$$

Nonetheless, for modeling purposes, it is quite difficult and inconvenient to implement wavelength-dependent properties and, thus, only scalar optical properties can be handled. This is the parameter used in the standard expression for the Beer-Lambert law as defined in Equation (5). *I*(0) is the integral of the spectral irradiance (*i.e.*, the incident heat flux from the cone) of the overall wavelengths at the sample surface. Note that Equations (3) and (5) are different only on the wavelength dependency.

(5) $$\text{I}\left(\text{x}\right)=\text{I}\left(0\right){\text{e}}^{-\text{A}\cdot \text{x}}$$

(6) $$\text{I}\left(0\right)=\int^{\text{​}}{\text{I}}_{\text{λ}}\left(0\right)\text{dλ}$$

Comparing Equations (4) and (5), and inserting Equation (6), it is possible to determine the scalar in-depth absorption coefficient as proposed in Equation (7).

(7) $$\text{A}=-\frac{1}{\text{x}}\cdot \text{ln}\left(\frac{\int^{\text{​}}{\text{I}}_{\text{λ}}\left(0\right){\text{e}}^{-{\text{A}}_{\text{λ}}\cdot \text{x}}\text{dλ}}{\int^{\text{​}}{\text{I}}_{\text{λ}}\left(0\right)\text{dλ}}\right)$$

This scalar absorption coefficient is dependent on the depth *x*. The part of the radiation where the spectrally resolved absorption coefficient is high will rapidly be absorbed near the surface. As the radiation penetrates the sample, the radiation spectrum will be distorted such that relatively more energy is concentrated to the wavelengths where the spectral absorption coefficient is lower. It should be noticed that the absorption coefficient A given by Equation (7), is *not* the local absorption coefficient at depth *x*. Instead, A is the parameter that gives the correct total irradiance at depth *x*, given by Equation (5), given an irradiation spectrum *I_λ_*(0) at the sample surface.

The choice of depth *x* in Equation (7) is somewhat arbitrary and will affect A, but we propose to use the depth where the total irradiation has decreased to e^−2^ ≈ 0.135. This depth is sometimes referred to as the skin depth or the penetration depth of the electromagnetic field. This absorption coefficient can be obtained by an iterative procedure using Equations (5) and (7), where A is calculated for a given *x* in Equation (7). Then, it is checked if *I*(*x*) ≈ 0.135 *I*(0) in Equation (5). Depending on the result, *x* is adjusted in Equation (7), *etc.*, until a self-consistent set of *x* and A is obtained.

The spectral transmittance through a thin sample is given by [35]:

(8) $${\text{T}}_{\text{sample},\text{λ}}=\frac{{\text{T}}_{\text{surface},\text{λ}}^{2}{\text{e}}^{-{\text{A}}_{\text{λ}}\text{d}}}{1-{\text{R}}_{\text{surface},\text{λ}}^{2}{\text{e}}^{-2{\text{A}}_{\text{λ}}\text{d}}}$$

where

*T*_sample,λ_ is the spectrally resolved transmittance through the entire specimen (measured);
*T*_surface,λ_ is the spectrally resolved transmittance through one surface of the specimen (not measured);
*R*_surface,λ_ is the spectrally resolved reflectance from one surface of the specimen (not measured);
*d*    is the thickness of the sample.

The denominator is close to unity and is approximated here to not differ significantly for similar sample thicknesses. Furthermore, the surface transmittance is independent of sample thickness. Therefore, by dividing the sample transmittance for two different sample thicknesses, the following relation is obtained in Equation (9). Equation (10) is used to calculate the scalar absorption coefficient A according to Equation (7).

(9) $$\frac{{\text{T}}_{\text{sample }1,\text{λ}}}{{\text{T}}_{\text{sample }2,\text{λ}}}={\text{e}}^{-{\text{A}}_{\text{λ}}\left({\text{d}}_{1}-{\text{d}}_{2}\right)}$$

or

(10) $${\text{A}}_{\text{λ}}=\frac{\text{ln}\left(\frac{{\text{T}}_{\text{sample }1,\text{λ}}}{{\text{T}}_{\text{sample }2,\text{λ}}}\right)}{{\text{d}}_{2}-{\text{d}}_{1}}$$

α_λ_ can be calculated from the reflectivity, *R*_λ_, and the transmittance, *T*_λ_, according to Equation (11). The reflectance from a thin sample can be expressed as in Equation (12) [35]. The scalar absorption coefficient *α* can thus be calculated using Equation (2) and the measurement of the reflectance and transmittance.

(11) $${\text{α}}_{\text{sample},\text{ λ}}=1-{\text{R}}_{\text{sample},\text{ λ}}-{\text{T}}_{\text{sample},\text{λ}}$$

(12) $${\text{R}}_{\text{sample},\text{λ}}={\text{R}}_{\text{surface},\text{λ}}\left(1+{\text{T}}_{\text{sample},\text{λ}}{\text{e}}^{-{\text{A}}_{\text{λ}}\text{d}}\right)$$

where

*R*_sample,λ_ is the spectrally resolved reflectance from the entire specimen (measured);
*R*_surface,λ_ is the spectrally resolved reflectance from one surface of the specimen (not measured).

### 2.6. Gasification Experiments

**Figure 2:**
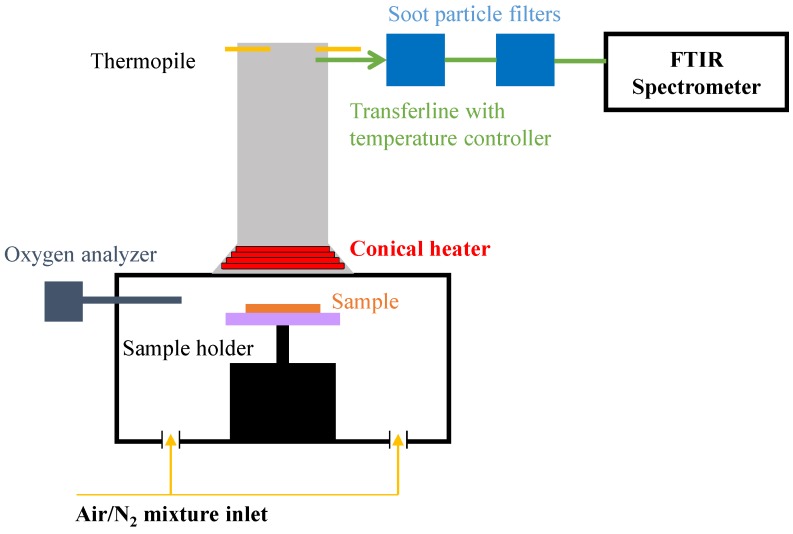
Schematic representation of CAMLC-FTIR apparatus.

A special setup of a mass loss cone from Fire Testing Technology Limited has been designed by isolating the cone heater and the sample in order to control the atmosphere. This controlled atmosphere mass loss cone (CAMLC) apparatus has been developed to study the influence of depleted oxygen environments (low ventilated fire) on the thermal decomposition of materials. A schematic representation of this apparatus is presented in Figure 2 and a more complete description can be found in [19]. The samples were wrapped in heavy gauge aluminum foil such that one face of the sample was exposed to the radiant heat flux. Each sample was placed on top of 50 mm of insulating firebrick (IFP) JM26^®^ from Morgan Thermoceramics and positioned 25 mm from the cone heater surface for all bench-scale tests. The fire parameters that can be measured in the CAMLC are limited to mass loss and mass loss rate.

To investigate the gases released during CAMLC experiments, the CAMLC apparatus was connected to a Fourier Transform Infra-Red (FTIR) spectrometer (CAMLC-FTIR) as shown in Figure 2. Using this device, released gases are analyzed online quantitatively and qualitatively. Gas picking pistol and transfer lines were provided by the M&C Tech Group; FTIR, Antaris™ Industrial Gas System, was provided by ThermoFisher Scientific. The transfer line between CAMLC and FTIR is 2 m long and was heated at 180 °C. To insure a constant temperature of the transfer line, two temperature controllers were installed. Before analyzing the gases by FTIR, soot particles were filtered off by two different heated filters (2 and 0.1 µm) that consist of glass fibers and ceramic, respectively. The FTIR gas cell was set to 185 °C and 652 Torr. The optical pathway is 2 m long and the chamber of the spectrometer is filled with dry air. FTIR spectra obtained using CAMLC-FTIR were treated using OMNIC™ software. To quantify gases, spectra have to be recorded at different concentrations for targeted gases and a quantification method has to be created using TQ™ Analyst software. To create a method, representative regions in the spectra of the selected gas have to be chosen and interactions with other gases have to be taken into account. Using CAMLC-FTIR the following gases can be quantified: water, carbon monoxide, carbon dioxide, acetic acid, acetone, methane, propane, ethane, and ethene. The developed method in our laboratory can also consider nitrogen monoxide, nitrogen dioxide, hydrogen cyanide, ammonia, and ethyne. Quantification is reproducible within ±10 %. CAMLC-FTIR experiments were performed in duplicate to ensure repeatability of the obtained results.

### 2.7. Pyrolysis Model Development

The commercial solver, Comsol^®^ Multiphysics, is able to solve non-steady energy and mass conservation equations accounting for chemical reactions described by a user-defined reaction mechanism. The reactions defined in the kinetic mechanism are governed by Arrhenius reaction rates defined as reported in Equation (13), where the reaction rate $\dot{r_{r}}$ is a function of temperature and the mass of reactant *m*_reactant_. The reaction presented in Equation (13) corresponds to a n^th^ order reaction. The activation energy (*E_r_*), pre-exponential factor (*A_r_*), stoichiometric coefficients (ν*^i^*) associated with species *i* and the order of the reaction (*n*) as defined in Equation (13) must be defined for each reaction.

(13) $$\dot{{\text{r}}_{\text{r}}}=-\frac{1}{{\text{υ}}^{\text{i}}}\frac{\partial {\text{m}}_{\text{i},\text{r}}}{\partial \text{t}}={\text{A}}_{\text{r}}\exp\left(\frac{-{\text{E}}_{\text{r}}}{\text{RT}}\right){\text{m}}_{\text{reactant}}^{\text{n}}$$

Mass and energy conservation equations are solved by Comsol^®^ Multiphysics assuming the heat exchange between the gases moving through the solid and the solid material occurs instantaneously. Mass transport inside the solid is assumed to be driven by the gradient of gas concentration. The general energy conservation equation in terms of temperature and the mass conservation equations are reported in Equations (14)–(16) respectively. Equation (15) is applicable for both Gas1 and Gas2 and different mass transfer coefficients can be applicable to these gases as their interaction with the media in which they diffuse can be different (affinity with the media, adsorption, chemical reactions or recombination, gas trapping, *etc.*). Equation (16) represents the mass conservation for solid species. The only differences between Equations (15) and (16) are the diffusion terms that represent the ability of the gaseous species to evolve from the material.

(14) $${\text{ρC}}_{\text{p}}\frac{\partial \text{T}}{\partial \text{t}}=\frac{\partial \left(\text{k}\frac{\partial \text{T}}{\partial \text{x}}\right)}{\partial \text{x}}+\overset{{\text{N}}_{\text{r}}}{\underset{\text{r}}{\sum }}\dot{{\text{r}}_{\text{r}}}{\text{H}}_{\text{r}}-\overset{{\text{N}}_{\text{g}}}{\underset{\text{i}}{\sum }}\left({\text{D}}_{\text{gas i}}\frac{\partial {\text{m}}_{\text{gas i}}}{\partial \text{x}}\right)\left({\text{C}}_{\text{p}}^{\text{gas i}}\frac{\partial \text{T}}{\partial \text{x}}\right)+\text{ε}\frac{\text{dI}(\text{x})}{\text{dx}}$$

(15) $$\frac{\partial {\text{m}}_{\text{gas i}}}{\partial \text{t}}=\overset{{\text{N}}_{\text{r}}}{\underset{\text{r}}{\sum }}\dot{\left({\text{ν}}_{\text{r}}^{\text{gas i}}.{\text{A}}_{\text{r}}\exp\left(\frac{-{\text{E}}_{\text{r}}}{\text{RT}}\right){\text{m}}_{\text{reactant},\text{ r}}^{\text{n}}\right)\;}-\frac{\text{d}}{\text{dx}}\left({\text{D}}_{\text{gas i}}\frac{\partial {\text{m}}_{\text{gas i}}}{\partial \text{x}}\right)$$

(16) $$\frac{\partial {\text{m}}_{\text{solid i}}}{\partial \text{t}}=\overset{{\text{N}}_{\text{r}}}{\underset{\text{r}}{\sum }}\left({\text{ν}}_{\text{r}}^{\text{solid i}}.{\text{A}}_{\text{r}}\exp\left(\frac{-{\text{E}}_{\text{r}}}{\text{RT}}\right){\text{m}}_{\text{reactant},\text{ r}}^{\text{n}}\right)\;$$

The energy conservation equation is solved taking into account conduction, heat generation from chemical reactions, in-depth absorption of the external heat source, and convection from adjacent elements. In gasification experiments using the CAMLC, no thermal shrinkage, intumescence, nor swelling of our material were observed, so the model is built in such a manner that these phenomenon are not considered. The mass conservation equation is solved taking into account the release of components due to chemical reactions and advection of gaseous components.

Boundary conditions are defined at the top and bottom surfaces of the computational domain to represent the conditions of the simulated experiment. As the sample material was heated, convective cooling to the atmosphere occurred with a convection coefficient of 10 W/m²/K, as determined in other studies [36,37,38]. The boundary conditions at the rear face of the specimen are represented by a 50 mm thick domain, representing the insulation, without thermal contact resistance with the studied material. The thermal properties associated with this insulation are given by the producer and are assumed to be constant with the temperature. The insulation has a density of 890 kg/m^3^, a heat capacity of 1100 J/g/K, and a thermal conductivity of 0.26 W/m/K. A constant ambient temperature of 300 K was assumed. The initial temperature is taken at 300 K and the initial amount of material corresponds to the mass of a plaque. The gaseous component, final residue, and intermediate species masses are thus set to 0 at the initial stage as the material consists exclusively of initial material (EVA/ATH). This model has proven to give similar results to both ThermaKin and FDS (version 6.1) in a previous study [19].

## 3. Results and Discussion

### 3.1. Determination of Input Data

**Figure 3:**
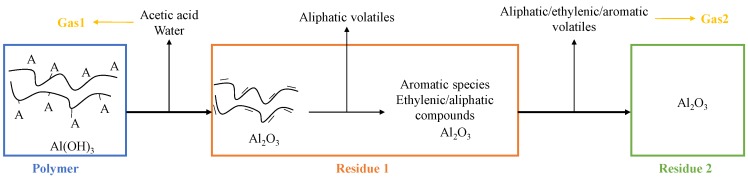
Decomposition pathway of an EVA/ATH formulation.

The first part of this work aims at investigating and developing several methods to evaluate the thermo-physical properties of EVA/ATH as a function of temperature. The decomposition of EVA and ATH is well described in the literature [32,39,40,41,42,43]: EVA starts to degrade under inert atmosphere around 300 °C in two steps, first releasing acetic acid and then hydrocarbons at around 450 °C. ATH dehydrates around 220 °C, releasing water and forming a ceramic residue made of alumina (Al_2_O_3_). In this study, the different decomposition (Figure 3) products will be defined as follows:

- Virgin material refers to the initial formulation of EVA/ATH.
- Residue1 is an intermediate specie and refers to alumina and to the remaining part of the EVA obtained after the deacetylation step. It can be found in the literature [32,39,40,44] that further hydrocarbons can be released from this material to form aromatic structures.
- Gas1 refers to the water released during the decomposition of ATH into alumina, and acetic acid from the deacetylation of the EVA.
- Residue2 refers to the alumina residue. EVA is assumed to be completely degraded.
- Gas2 is a mixture of hydrocarbon gases evolved during the decomposition of the EVA.

#### 3.1.1. Kinetic Analysis and Determination of the Enthalpies of Reaction

**Figure 4:**
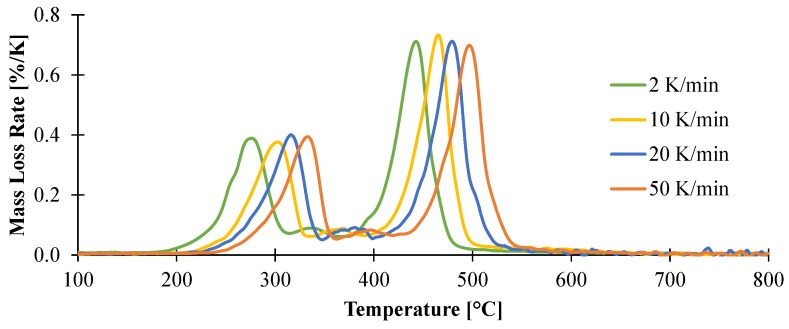
Mass loss rate collected in TGA experiments carried out in nitrogen at a heating rates of 2, 10, 20, and 50 K/min.

The thermal decomposition of the material has been studied at different heating rates from 2 K/min to 50 K/min. In these conditions, it is expected to capture the overall decomposition of the material in a wide range of conditions (from slow to high heating rates mimicking different fire scenarios). For all the heating rates, the material exhibits two main apparent steps of decomposition evidenced by the two peaks of mass loss rate (MLR) curves as shown in Figure 4. The first peak of decomposition, corresponding to the formation of an intermediate species (Resiude1) and the release of Gas1 as defined in Figure 3, exhibits an onset temperature from 200 to 240 °C for all the heating rates and its peak of mass loss rate (pMLR) can vary from 250 to 320 °C depending on the heating rate. The second decomposition step, corresponding to the decomposition of the remaining polymer part of the formulation and the release of Gas2 as defined in Figure 3, is more intense than the first one and appears at 400 °C for the lowest heating rate with pMLR between 440 and 510 °C.

**Figure 5:**
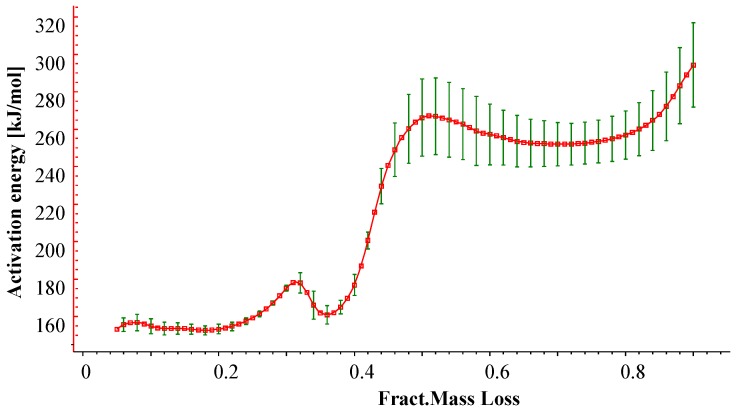
Activation energy as a function of fractional mass loss according to Friedman analysis.

Before starting any fitting procedure, it is necessary to define a reaction model (combination of reactions) and to preset starting values for the kinetic parameters. A convenient approach is to use model-free analysis as a preliminary step of the kinetic analysis. A model-free analysis (Friedman analysis) provides the plot of the activation energy *versus* the fractional weight loss as shown in Figure 5. For EVA/ATH formulation, it reveals that the plot of activation energy exhibits a simple shape with two consecutive and distinct steps as shown in Figure 5. The first one, at a degree of conversion lower than 0.4, exhibits an activation energy around 160 kJ/mol, while at a higher degree of conversion, the activation energy reaches a plateau at 260 kJ/mol. Then these results were used as initial parameters and nonlinear regression was carried out in order to find the best fit between experimental and simulated data for all the studied heating rates. 

**Figure 6:**
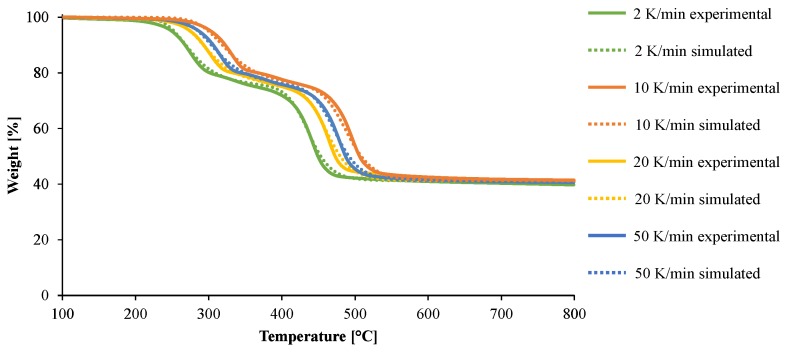
Simulated (dotted lines) and experimental (straight lines) weight loss curves under nitrogen at heating rates of 2, 10, 20, and 50 K/min.

According to the previous analysis, the decomposition of EVA/ATH is modeled using two successive steps with n^th^ order functions and the optimum parameters are shown in Table 1. This kinetic model is chosen as it allows a good description of the decomposition of the material and provides the best quality of fit between the computed and the experimental TG curves. The comparison between the experimental and the simulated data is shown in Figure 6. It is noteworthy that the simulated and experimental data show a good agreement. In particular, the simulated pMLR for both the first and second steps of decomposition at each heating rate is predicted with an error of less than 5% (not shown). It is also worth noting that the values found for the activation energies are consistent with those already reported for EVA [32,39]. Indeed, for the first step of decomposition of EVA/ATH formulation corresponding to the deacetylation of the EVA, Rimez *et al.* [39] found a pre-exponential factor log A of 12.7 against 12.9 in this study and an activation energy of 163.8 kJ/mol for EVA (with a vinyl acetate contents lying between 9 and 33 wt %) against 162 kJ/mol in this study. In the first decomposition step, the dehydration of ATH must also to be taken into account. Cérin *et al.* [32] found a value of 113.6 kJ/mol and a log A of 8.73 but associated the decomposition of the ATH to a first order Avrami Erofeev reaction in the case of an EVA/ATH formulation containing 60 wt % of vinyl acetate. The values obtained for our EVA/ATH are higher than those associated with ATH dehydration but similar to those reported for deacetylation. Note that the kinetic parameters used in this study are not associated with ATH dehydration or EVA deacetylation but more generally describe the decomposition step from the pristine material to the alumina/polyene system as defined in Figure 3. Note also that the differences may come from the different reaction models used in the different studies: first order Avrami Erofeev in [32] and the second-order reaction in this study. Rimez *et al.* also [39] studied the decomposition of the remaining polyene resulting from the deacetylation of the EVA. A pre-exponential factor log A of 16.3 and an activation energy of 260.7 kJ/mol in the case of the formation of aliphatic volatiles were found. These values are similar to our results corresponding, respectively, to 17.1 for log A and 268 kJ/mol for activation energy for our material. Even if the real reactions of the system are too complex to be characterized in any fundamental way, the reactions are described as pseudo (or lumped) species which are themselves complex materials or mixtures.

**Figure 7:**
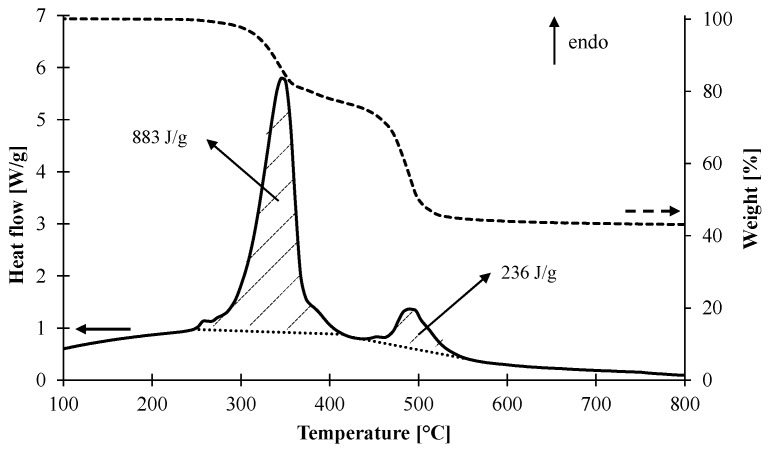
DSC signal of the decomposition of EVA/ATH (straight line) and TG signal (dotted line).

The enthalpies of decomposition were measured with simultaneous thermal analysis under nitrogen and are reported in Table 1. ATH is known to undergo an endothermic reaction: Al(OH)_3_ releases water, forming a ceramic residue made of alumina (Al_2_O_3_). We found experimentally that the first step of decomposition exhibits an endothermic decomposition with an endothermic enthalpy of H_r1_ of 883 J/g as shown in Figure 7. The second reaction associated with the pyrolytic decomposition of the polymer is still endothermic but its enthalpy of reaction H_r2_ is only 236 J/g. 

**Table 1:** Kinetics of decomposition and heats of reactions assuming a two-step mechanism.

Reaction	*A_r_* (1/s)	*E_r_* (kJ/mol)	Reaction Order	*Hr* (J/g)
1	8.71 × 10^12^	162	3	883
2	1.23 × 10^17^	268	2	236

#### 3.1.2. Determination of Thermal Conductivity of the Material as a Function of the Temperature

**Figure 8:**
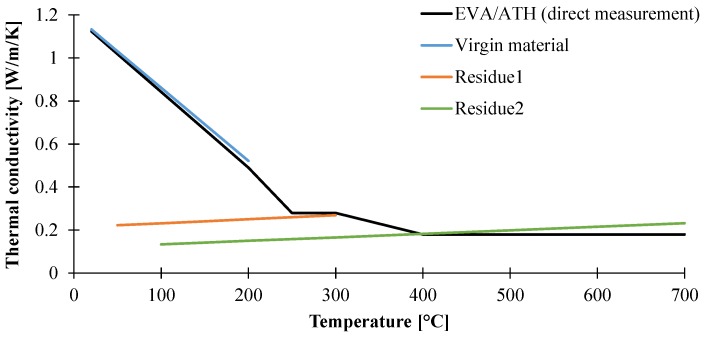
Comparison of thermal conductivities of EVA/ATH and EVA/ATH decomposition species as a function of temperature.

The thermal conductivity of the material was investigated using a Transient Plane Source method from ambient temperature to 700 °C. The thermal conductivity was first directly measured and it was shown that the thermal conductivity steadily decreases from ambient temperature to 250 °C as shown in Figure 8. Above this temperature, the material contains alumina (Al_2_O_3_) which can act as a thermal barrier and leads to a lower conductivity. From 300 to 400 °C, the thermal conductivity of the material remains constant, equaling 0.28 W/m/K. Then, above 400 °C, the thermal conductivity remains constant at 0.18 W/m/K up to 700 °C. The decrease of thermal conductivity at 400 °C can be attributed to the complete decomposition of the remaining polymer and thus modifies the insulative properties of the ceramic residue. On each temperature range, the thermal conductivity of the material was then extrapolated with linear function and can be expressed as presented in Table 2. 

**Table 2:** Thermal conductivity of EVA/ATH as a function of temperature.

Thermal Conductivity (W/m/K)	Temperature Range (°C)
1.17–3.21 × 10^−3^ T	Ambient–250
0.28	250–400
0.18	400–700

As far as we know, there are few indications in the literature about the expected range of thermal conductivity at high temperatures for flame-retardant materials and especially for ATH-based formulations. Nonetheless, Hoffendahl *et al.* [45] studied the thermal conductivity of a 60 wt % vinyl acetate EVA flame retarded with 65 wt % of ATH in combination with melamine derivatives. They found that the ambient thermal conductivity was in the range of 0.8–0.9 W/m/K and steadily decreased to around 0.3 W/m/K in the range 350–400 °C. At temperatures higher than 400 °C, the thermal conductivity of the residue formed by the decomposition of the EVA/ATH system with or without melamine, melamine borate, or melamine phosphate was in the range 0.3–0.2 W/m/K. Those values are thus in agreement with our study.

**Figure 9:**
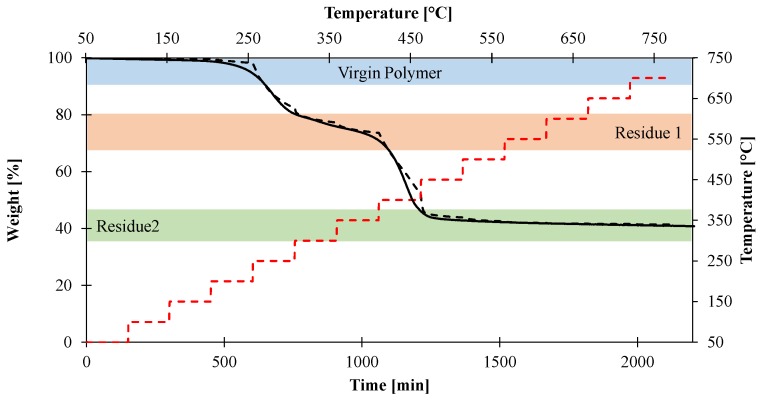
Isothermal TGA experiments (dotted line) and dynamic TGA experiment at 2 K/min (straight line) performed in nitrogen atmosphere.

In order to further investigate the thermal conductivity of the material, a second approach was followed. Indeed, even if we are able to express the thermal conductivity of our material as a function of temperature, parameters used in the pyrolysis model are generally described both as a function of temperature and composition. Indeed, as previously explained, EVA/ATH material can be described using three independent species: the virgin material, an intermediate residue formed of alumina and polyenes (Residue1), and the final residue (Residue2) consisting exclusively of alumina. The domain of stability and the presence of these three species depends on the heating conditions. Indeed, it is obvious that if the onset temperature of decomposition of EVA/ATH at 2 and 50 K/min moves from 200 to 240 °C, it is not possible to define a temperature corresponding to the transition from one product to another. As thermal conductivity measurements were carried out using isothermal stepped experiments and because a steady state is required for the measurement (not dynamic heating ramps as in classical TGA experiments), we had no indications about the presence of the species at each steps. In order to obtain more information, in particular about the weight loss, the decomposition of the material was studied using specific stepped TGA experiments mimicking temperature stepping in heat conductivity measurements. Thus, the material underwent isothermal steps of 150 min every 50 °C from 50 to 700 °C. The duration of the steps was chosen with respect to the measurement time required when evaluating thermal conductivity. The results are shown in Figure 9. The pristine EVA/ATH does not decompose before the isothermal step 200 °C. The thermal conductivity determined in the range 20–200 °C could thus be attributed to the virgin polymer. Then, at higher temperatures, the intermediate species (Residue1) corresponds to a residual weight lying between 80 and 75 wt % (temperature range between 300 and 350 °C). The final residue (Residue2) is defined when the organic material is completely decomposed, corresponding to a residual weight of 40–45 wt % (temperature range between 500 and 700 °C).

Based on those results, additional thermal conductivity experiments have been performed in order to determine the thermal conductivity as a function of both temperature and composition. For the determination of the thermal conductivity of the pristine EVA/ATH, temperatures higher than 200 °C were not considered. For the determination of the thermal conductivity of the intermediate species (Reside1) as a function of the temperature, the material underwent an isothermal step of 150 min at 300 °C without any measurements. The objective was to form Residue1. Then, the system was cooled down to 100 °C and the measurements were done from 100 to 350 °C with an increment of 50 °C. For the assessment of the thermal conductivity of the final residue (Residue2) as a function of temperature, a similar procedure was carried out: the system underwent an isothermal step of 150 min at 500 °C and was cooled down to 100 °C. The thermal conductivity was then measured from 100 to 700 °C with 100 °C increments. Using those procedures, it was possible to evaluate the thermal conductivity both as a function of temperature and composition as presented in Table 3. A comparison between direct measurement and both composition- and temperature-dependent measurements is also presented in Figure 8. It is observed that the thermal conductivity of the EVA/ATH shows a good agreement with the pristine EVA/ATH. It can also be noted that, in the range 250–300 °C, the direct measurement of the thermal conductivity of EVA/ATH as a function of temperature is consistent with that of the intermediate residue (Residue1). Similar results are obtained at temperatures higher than 400 °C with the thermal conductivity of the final residue (Residue2).

**Table 3:** Thermal conductivity of EVA/ATH decomposition species as a function of composition.

Thermal Conductivity (W/m/K)	Specie	Temperature Range
1.20 – 3.39 × 10^−3^ T	Virgin EVA/ATH	<250 °C
0.21 + 1.89 × 10^−4^ T	Residue1	<350 °C
0.11 + 1.63 × 10^−4^ T	Residue2	>400 °C

Based on those results, the thermal conductivity can be implemented in two different ways in the pyrolysis model: either the direct measurement on the EVA/ATH, or a linear combination of the thermal conductivities of the pristine EVA/ATH, the intermediate specie (Residue1), or the final residue (Residue2). This last approach is assumed to be more precise and more realistic, and is generally used in pyrolysis modeling studies. Numerically, the thermal conductivity is a function of the thermal conductivities of the species and of the kinetics of decomposition. This can, however, lead to additional errors if the kinetics of decomposition cannot capture the entire heating rate range at which the material is submitted. On the contrary, using the direct measurement on the EVA/ATH cannot dynamically capture the process of decomposition. Nonetheless, its characterization is easier (in both time and procedure) than that of the thermal conductivities of each species, in particular if more than two species (generally the initial polymer and the residue left by the decomposition) are observed.

#### 3.1.3. Determination of Heat Capacity of the Material as a Function of the Temperature 

**Figure 10:**
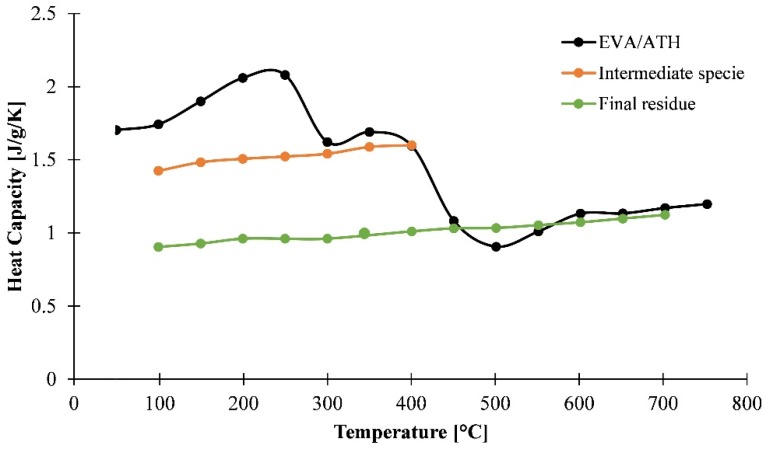
Heat capacity of EVA/ATH as a function of temperature assessed through mDSC steps.

The heat capacity of the EVA/ATH formulation was investigated using modulated DSC coupled with a TGA. This was made possible by the resolution of the STA analyzer used and by increasing the mass of the sample. As shown in Figure 10, the heat capacity increases steadily before decomposition (<220 °C) from 1.68 to 2.04 J/g/K at 200 °C and remains constant at around 2.07 J/g/K until 250 °C. Then, the heat capacity of the material decreases to 1.5 J/g/K when the mass stabilizes around 75 wt % residual weight. In the range 300–400 °C, corresponding to the range of stability of Residue1, the heat capacity ranges between 1.58 and 1.68 J/g/K. Then the material undergoes its final decomposition step and Residue2 exhibits a heat capacity which ranges from 0.89 to 1.18 J/g/K between 500 and 800 °C. Those values are consistent with the heat capacity of charring and non-charring polymers found in the literature [22,36,46], but also with the heat capacity already reported for a polyethylene flame retardant with a high content of ATH [19]. On the other hand, EVA is a non-charring polymer and leaves no high temperature residue, but EVA/ATH yields to an alumina ceramic at high temperatures. The values obtained at high temperatures should thus be compared to these of this ceramic. It is found that the values of the heat capacity commonly reported in the literature for alumina [24,25] range between 0.8 and 1.24 J/g/K in the range 25–727 °C, which is consistent with our results

**Figure 11:**
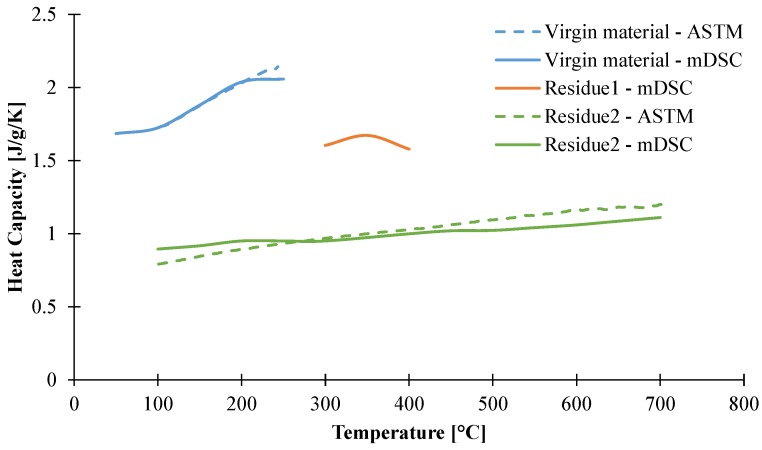
Heat capacity of EVA/ATH decomposition species as a function of temperature assessed through mDSC steps and dynamic heating ramps.

As it was done for thermal conductivities, the heat capacity was also determined by a stepped method. The same procedure as that for the thermal conductivity measurements was used. The results, shown in Figure 11, are consistent with the previous analysis. The intermediate species exhibits a linearly increasing heat capacity from 1.4 J/g/K at 100 °C to 1.58 J/g/K at 400 °C. The heat capacity of the final residue also exhibits an increasing behavior as it starts from 0.89 J/g/K at 100 °C to 1.11 J/g/K at 700 °C. Between 300 and 400 °C for the intermediate species and at temperatures higher than 500 °C for the final residue, the measurements of the species-dependent heat capacities are in agreement within 5% standard deviation, with the direct measurement on EVA/ATH.

Another experimental approach, based on the ASTM E1269-11 [16] standard, has been used to investigate the heat capacity of our material. Namely, the material underwent a 20 K/min heating rate ramp from 50 to 800 °C preceded and followed by 15 min isothermal steps. Those steps are necessary for heat flow signal equilibrium and the heating rate is recommended by the standard to ensure the best signal [16]. 

Before decomposition of the material, this method is in agreement with mDSC measurements as the heat capacity is 1.82 J/g/K at 100 °C and 2.20 J/g/K at 200 °C using this method (against 1.78 J/g/K at 100 °C and 2.04 J/g/K for mDSC measurements), as shown in Figure 11. It is noteworthy that this procedure does not allow us to have information about the heat capacity of the intermediate species, as the two decomposition steps are successive and the heat of the reactions are overlapping in the region where the intermediate species is stable; thus, it is not possible to determine a heat capacity. Even if it gives less information than the mDSC measurements, this method is still useful as it is less time-consuming compared to isothermal mDSC measurements and also gives us access to the heat of reactions. Nonetheless, it should be noticed that ASTM E1269 is assumed to be valid only if the mass change is lower than 0.3 wt % and this technique is thus applicable only before decomposition or on the final residue. Thus, the residue was collected at the end of the experiment and cooled to ambient temperature. Then it underwent a heating rate of 20 K/min and the heat capacity was measured according to ASTM E1269. As shown in Figure 11, the heat capacity assessed by the ASTM method is consistent with mDSC measurements, as an increasing heat capacity was measured. The heat capacity varies from 0.73 J/g/K at 100 °C to 1.23 J/g/K. The results using the ASTM method and isothermal mDSC steps are in agreement with each other, but also with the values of alumina found in the literature within 10% standard deviation [24].

#### 3.1.4. Determination of Heat Capacity of the Decomposition Gases 

For modeling purposes, it is of interest to know the heat capacities of the decomposition gases as they can transport energy when diffusing inside the decomposing material. This is mathematically described by the term $\overset{N_{g}}{\underset{i}{\sum }}\left(D_{\text{gas }i}\frac{\partial m_{\text{gas}\;i}}{\partial x}\right)\left(C_{p}^{\text{gas }i}\frac{\partial T}{\partial x}\right)$ in Equation (14) and the amount of energy that gases can transport is directly proportional to the mass flow inside the material but also to the heat capacity of these gases $C_{p}^{g}$. As the quantity and nature of those gases can influence the temperature distribution inside the material, it is important to have more qualitative and quantitative information about them. The identification of gaseous decomposition products can be obtained through TGA experiments coupled with FTIR analysis. This was shown to be very effective when investigating the thermal decomposition of EVA filled with various metal oxides [45,47]. In this work, it was decided to use a controlled-atmosphere mass loss calorimeter coupled with a calibrated FTIR spectrometer (CAMLC-FTIR) to detect and to quantify the products of the gasification of EVA/ATH in conditions similar to the cone calorimeter experiments. This approach has proven to be effective for the quantification and has already been used in the case of the investigation of flaming cone calorimetry experiments [45,47,48].

**Figure 12:**
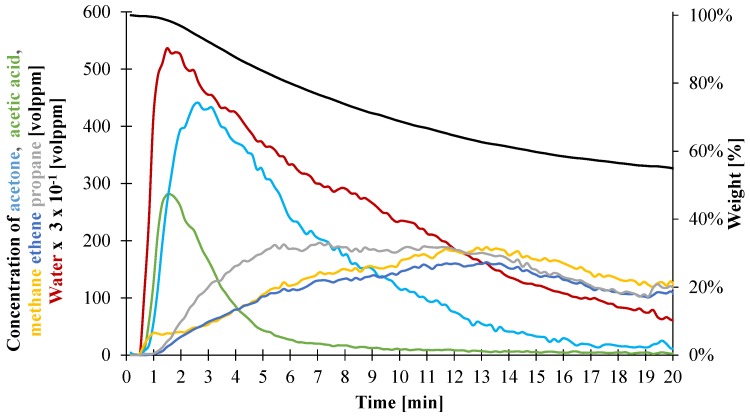
Average mass measured and concentrations of each gas released during CAMLC-FTIR experiments on 3 mm thick EVA/ATH plaque exposed to 50 kW/m².

CAMLC-FTIR experiments have been performed with an oxygen level as low as 2 vol % in the gasification enclosure. During the whole duration of the test, the following gases are mainly detected: water, acetone, acetic acid, and hydrocarbons compounds such as propane, methane, and ethene. The results are presented in Figure 12. In the first part of the experiment, the material simultaneously releases acetic acid, coming from the deacetylation of the vinyl acetate part of the polymer and water, from the dehydration of the ATH. It is noteworthy that the peaks of water and acetic acid emission occurs concurrently with the peak of mass loss rate (pMLR) at around 1.8 min. Both gases exhibit the same behavior: the gases start to evolve when the material degrades at 0.5 min and increase until reaching a peak at around 1.8 min, and then decrease during the duration of the test. Acetone is also detected and exhibits a similar behavior than acetic acid and water but its peak is delayed to 3 min. As already reported by Witkowski *et al.* [42], acetone comes from the catalytic reaction of acetic acid on alumina. When exposed to the incident heat flux, the material starts to degrade, releasing water and acetic acid and forming alumina and polyenes. As an alumina layer is developing on the top of the decomposing material during the test, the acetic acid evolving from the material can then react at this surface and form acetone. The delay between the peak of acetone and acetic acid release can be due to the time required for the alumina layer to be formed in a sufficient quantity to promote the formation of acetone or the time required for this layer to reach the temperature to activate the catalytic process. A small amount of methane, lower than 60 vol ppm, is also observed at that stage. After 2 min, a second stage of decomposition can be observed during which hydrocarbon compounds are released in larger quantities. During this step, acetic acid, acetone, and water release are decreasing as the decomposition front reaches the bottom of the cone plaque, and thus those fuels cannot be formed. More precisely, propane and ethene are detected alongside methane already identified in a lower amount in the first stage of decomposition. These three gases start to evolve around 2 min and their concentrations steadily increase before reaching a maximum around 160 vol. ppm between 11 and 15 min. It can be noted that propane concentration increases more rapidly than methane and ethene as it reaches 160 vol ppm after 5 min of experiment. From 10 min until the end of the experiment, a volumetric ratio of 1/1/1 between methane, ethane, and propane can be observed.

In order to obtain to the heat capacity of the decomposition gases, we decided to evaluate the total amount of evolving gases for each product. The results are presented in Table 4. It is interesting to note that, in the first step of decomposition (Gas1 as defined in Figure 3), water is mainly released. On the contrary, in the second stage of the decomposition (Gas2 as defined in Figure 3), the hydrocarbon compounds are released in similar quantities. Information about the heat capacity of each individual gas has been found in the literature [49]. Nonetheless, the quantification of the gases in the gasification experiments has been done in terms of volumetric concentrations, but the pyrolysis model is solved *versus* mass. The volumetric ratio of the different individual gases has thus to be translated to mass ratio. Assuming the ideal gas law is valid for all the quantified gases, it is possible to define the weight ratio between two gases as the ratio between the yields of the volumetric quantification obtained via FTIR and the molar masses of the studied gases. Mass ratio between acetone, acid acetic, and water for the first decomposition gas and propane, ethane, and methane for the second decomposition gas are then calculated and the composition of the decomposition gases is presented in Table 4.

**Table 4:** Total volumetric concentration of gases and mass composition of Gas1 and Gas2.

	Gas1			Gas2		
	Acetic Acid	Acetone	Water	Ethene	Propane	Methane
Total Gas Evolved (vol. ppm)	837	2936	151,337	2588	3110	3017
Mass ratio of evolved gases	2.0 × 10^−2^	6.0 × 10^−2^	1.0	1.5	2.8	1.0
Composition in individual gas (wt %)	1.7	5.8	92.5	28.1	53.1	18.8

**Figure 13:**
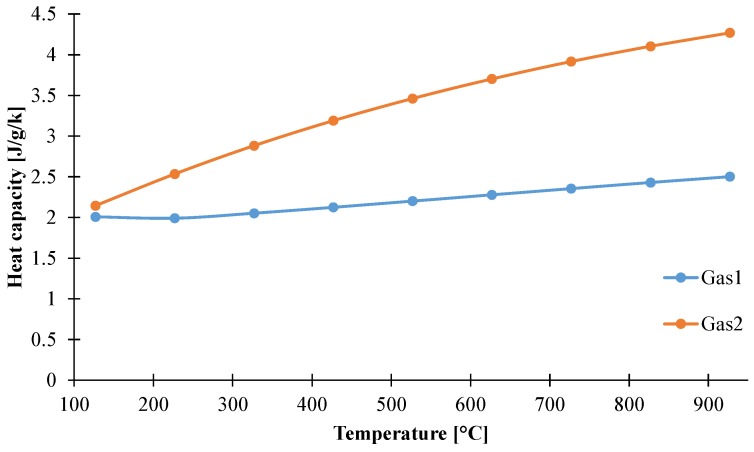
Heat capacities of Gas1 and Gas2 as a function of temperature.

It can be noticed that, during the first decomposition step, mainly water is released. This is consistent with the composition of the material composed at 65 wt % of ATH which itself releases water when decomposing. Due to the difference in molar masses, Gas2 is composed of more than 50 wt % of propane. Based on those compositions, and using heat capacity values from the literature [49], it is possible to evaluate the heat capacity of the decomposition gases. The results are presented from 100 to 900 °C in Figure 13.

It can be noted that the heat capacity of Gas1 is nearly constant. It varies following a gradual increase from 2.0 J/g/K at 127 °C up to 2.5 J/g/K at 927 °C. Interestingly, the heat capacity of Gas1 is close to that of water, as it is only higher than 10% during the overall range of temperature. On the contrary, the heat capacity of Gas2 is constantly increasing and greatly varies in the considered range of temperature. It starts at 2.15 J/g/K at 127 °C and rises up to 4.26 J/g/K at 927 °C. The relatively high value for the heat capacity of Gas2 compared to Gas1, or even of the condensed phase species previously measured, is due to the composition in hydrocarbon volatiles and their relative high heat capacity compared to water [49]. It can be noted that the values obtained are different to other heat capacities of gases reported in other studies dealing with pyrolysis modeling. For example, Li *et al.* [36] used the temperature-averaged heat capacity of gaseous hydrogen chloride when modeling pyrolysis of PVC. Hydrogen chloride was chosen assuming that poly-vinyl chloride releases more than 80 wt % of this gas. To model the pyrolysis of other polymer such as poly-carbonate, high impact poly-styrene, or polyoxymethylene, Li *et al.* [13] used the average heat capacity of linear C_1_-C_8_ hydrocarbon gases between 400 and 500 K and a heat capacity for the gaseous component of 1.8 J/g/K was obtained and used for further modeling. Even if the heat capacities of C_1_–C_8_ linear hydrocarbons are very similar, as they are in the range 2–2.4 J/g/K at 100 °C and exhibit an increasing behavior up to 2.7–3.4 J/g/K at 900 °C [49], other compounds such as alkenes and aromatic volatiles can be released and should also be considered. However, limited values for those gases are found in the literature. Moreover, the temperature increase in gasification experiments can go up to 600 K [13] and it can be questionable to use 400–500 K averaged values and not temperature-dependent values. All those assumptions can be acceptable if the transport of the gaseous component out of the materials is fast, but it leads to higher uncertainties if the diffusion of the decomposition gases inside the material is slower. Indeed, gases can transport and evacuate energy from the decomposition front out of the material. 

#### 3.1.5. Determination of the Optical Properties

**Figure 14:**
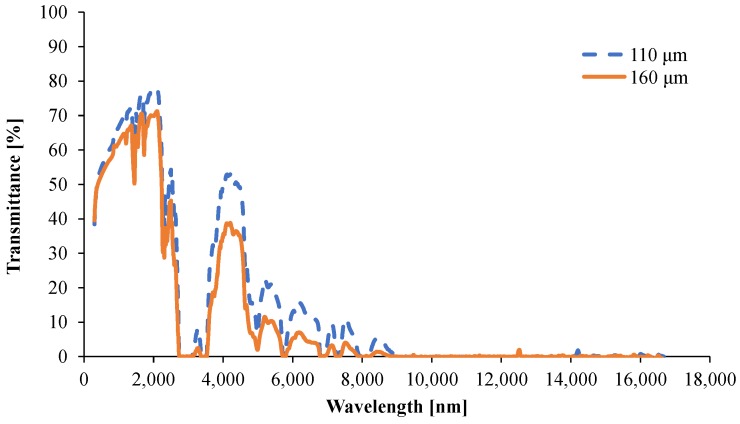
Spectrally resolved transmittance for 110 and 160 μm films.

**Figure 15:**
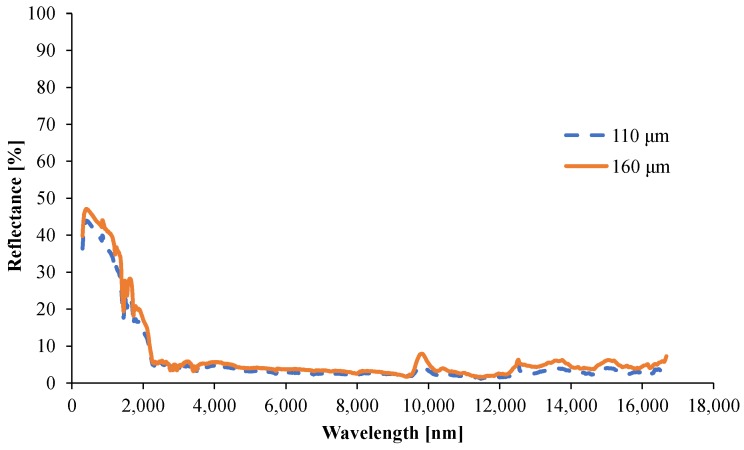
Spectrally resolved reflectance for 110 and 160 μm films.

The transmittance spectra for the two samples with 110 μm and 160 μm thickness are shown in Figure 14, and the reflectance spectra are shown in Figure 15. The spectrally resolved absorption coefficient, *A*_λ_, is obtained from Equation (12). However, the transmittance is near zero for certain wavelength intervals, meaning that the signal-to-noise ratio of the measurement is too low, or zero, so the spectral absorption coefficient cannot be determined at these wavelengths. In this work, the simple and very approximate approach from Witkowski *et al.* [19] is set at *A*_λ_ = 50,000 m^−1^ for the spectral ranges when the signal-to-noise ratio is too low for the considered spectral ranges. Using this filter and using Equations (7) and (9), a total absorption coefficient *A* = 8,200 m^−1^ is obtained, with a skin depth of 250 μm. Finally, these results together with the reflectance spectrum for the 160 μm samples, and Equations (4), (11), and (12), give a scalar absorptivity of 95%.

#### 3.1.6. Conclusion about the Characterization of the Inputs Data

In this first part, experimental techniques were developed to determine input data needed to model the pyrolysis of EVA/ATH. In particular, different approaches were developed to determine the heat capacity and thermal conductivity: (1) “direct” methods, consisting of determining the values of heat capacity and thermal conductivity in one experiment; (2) composition-dependent techniques considering the various steps of decomposition of the material (virgin, Residue1, Residue2, as defined in Figure 3). For the latter, two approaches can be followed: either the parameter is determined *versus* temperature, or it can be determined at ambient temperature. Considering the composition-dependent value for heat capacity or thermal conductivity, the input is then considered as a linear combination of each species weighted by their actual content as defined in Equation (17), where *X* is a property such as heat capacity or thermal conductivity, for instance, and can be either temperature-dependent or a constant measured at ambient temperature.

(17) $$\text{X}=\frac{{\text{M}}_{\text{virgin}}}{{\text{M}}_{\text{total}}}{\text{X}}_{\text{virgin}}+\frac{{\text{M}}_{\text{intermediate}}}{{\text{M}}_{\text{total}}}{\text{X}}_{\text{intermediate}}+\frac{{\text{M}}_{\text{residue}}}{{\text{M}}_{\text{total}}}{\text{X}}_{\text{residue}}$$

### 3.2. Modeling of the Gasification of EVA/ATH

**Figure 16:**
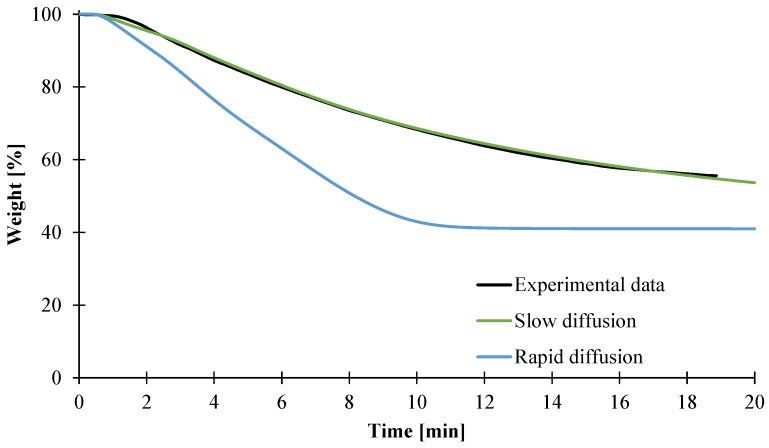
Experimental and predicted curves assuming instantaneous release of the gases or slow release of gases.

In this part, the numerical model is used to generate mass loss and temperature curves obtained for gasification experiments performed at 50 kW/m² on 100 × 100 × 3 mm^3^ plaques. In a first step, we used temperature- and composition-dependent heat capacity and thermal conductivity. It is generally found in the literature that the decomposition gases are released rapidly [46,50,51]. This is mathematically described by a mass transfer coefficient (*D_gas_* in Equation (14)) of 2 × 10^−5^ m²/s. Using this assumption does not allow us to predict the mass loss curve well. Indeed, it is obvious that assuming fast diffusion of the gases in the model over-predicts the mass loss, and the mass loss rate as the predicted curve is always below the experimental curve (Figure 16). Moreover, the final mass loss after 20 min is assumed to be 41 wt %, while it was found experimentally to be 56 wt %. Slower diffusion must therefore be considered and lower *D* values are relevant. Hoffendahl *et al.* [45] investigates the thermal decomposition of EVA/ATH together with melamine borate as a synergist in a mass loss cone calorimeter under 35 kW/m² incident heat flux. The bottom and front of a 3 mm thick plaque were analyzed using ^13^C and ^27^Al solid-state NMR. As soon as the material ignites (starting pyrolytic decomposition of the material), it was found that it took around 400 s for the ATH to be turned into alumina at the bottom of the plaque. Similarly, it was found that around 600 s were necessary for the polymer part of the material to decompose at the backside of the plaque. Even if the material studied here is different (lower VA content and lack of synergistic melamine derivative), it can be assumed that our EVA/ATH formulation will decompose in a similar manner or even faster, as the incident heat flux is higher. This would mean that in the first 10 min of the CAMLC experiments, the material would completely decompose, and thus no mass loss would be observed afterwards, which is consistent with the predicted mass loss curve and the kinetics of decomposition found in this study assuming a mass transfer coefficient of 2 × 10^−5^ m²/s (Figure 16). Moreover, as seen previously, acetic acid turns into acetone when diffusing in aluminum oxide [42]. It was found in the literature that acetic acid adsorbs onto alumina oxide when reacting to form acetone [52,53,54]. This would also promote the assumption of slower release of the decomposition gases. Based on those results, it was thus decided to tune the mass transfer coefficient of each released gas in order to fit the experimental mass loss. This fitting procedure is a way to estimate those mass transfer coefficients as they are the sole and only unmeasured parameter in the model. A mass transfer coefficient for Gas1 of 13 × 10^−9^ m²/s and a second mass transfer for Gas2 were then estimated at 4 × 10^−9^ m²/s in order to fit the last part of the mass loss curve. Note that those parameters are in the same range of magnitude as the mass transfer coefficient used by Witkowski *et al.* [19] (8 × 10^−9^ m²/s), validating the approach and the values found in this study. This approach was also used by Statler *et al.* [55] for the modeling of an intumescent PC, but a value of 1.8 × 10^−6^ m²/s was used then.

**Figure 17:**
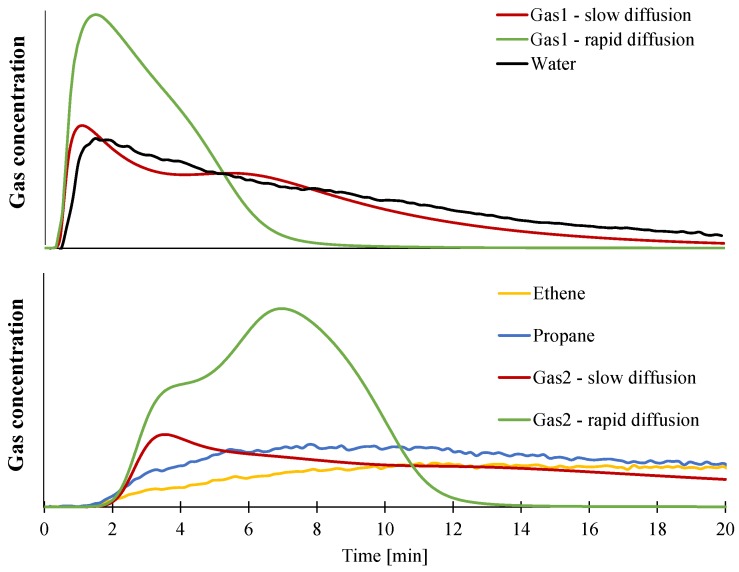
Experimental and predicted gas release assuming slow or rapid diffusion of gases.

As described previously, it is possible to collect temporally resolved gas concentration profiles during CAMLC-FTIR experiments. Two main gases are evolved: Gas1, which corresponds to approximately 95% of water, and Gas2, which correspond to hydrocarbon compounds. The mass flux of both Gas1 and Gas2 have thus been simulated assuming either rapid diffusion using a mass transfer coefficient of 2 × 10^−5^ m²/s or assuming slow diffusion as defined previously, and results are presented in Figure 17. They show that the release of the decomposition products is well predicted using the assumption of slow diffusion of the gases. Indeed, with a high *D_gas_* value, Gas1 exhibited a peak of release around 2 min, but too much of the gases is released and no more water is released after 6 min. The difference between the experimental and predicted time to peak can be attributed to the acquisition time of the FTIR spectrum (one spectra consists of 20 scans). The prediction of the release of hydrocarbon compounds is also significantly improved when assuming a slow release of gases. Indeed, the rate of release of Gas2 is close to these of ethene and propane until 6 min. Fitting the experimental gases that are the most representative of the gaseous component inside the model (water for Gas1 and ethene, propane or methane for Gas2) validates the change of mass transfer coefficient for these two gases.

**Figure 18:**
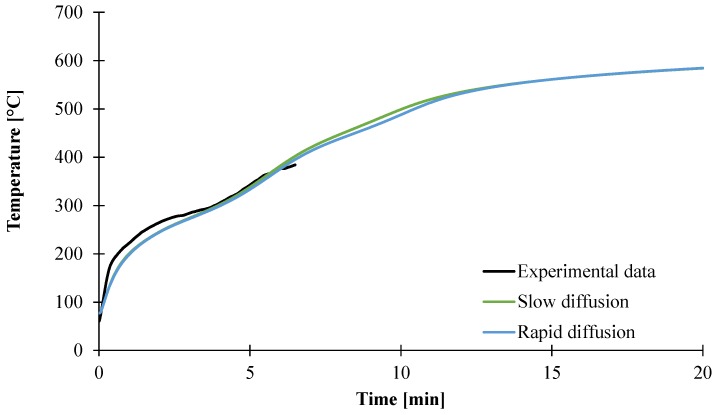
Experimental and predicted temperature increase at the backside of a plaque.

At the exception of the mass transfer coefficient, all the input data have been measured as shown previously. In order to validate these data (including *D_gas_* values), the temperature profile at the backside of the plaque was predicted using the model and compared with experimental data in the case of the slow diffusion of decomposition gases as well as the fast diffusion (high *D_gas_* values). Note that only the first six minutes of the experiment data are presented, as the thermocouples molded at the backside were not retained anymore after that time. As shown in Figure 18, a good agreement between predicted and experimental data is obtained assuming slow diffusion but also fast release of gases. Before 5 min, both predicted temperature curves are similar. Also, after the 12 min experiment, when the temperature reaches a plateau around 580 °C, the prediction shows a difference of less than one degree Kelvin. Between 5 and 12 min, the predicted temperature curve assuming slow diffusion is 10 °C higher than if assuming fast release of the gases.

It is interesting to note that, in this study, the energy removed by the diffusion of the gases does not play an important role in the temperature profile. Indeed, by integrating the heat absorbed by chemical reaction and by gas diffusion (which is directly proportional to mass flow and mass transfer coefficient, and also heat capacity of the gases), it is possible to evaluate the relative importance of each phenomena on the energy generation or absorption. It was found that around 68%–75% of the energy is absorbed by the chemical reaction corresponding to the dehydration of ATH. On the contrary, only 6%–12% of the energy absorbed is attributed to the heat evacuated by the diffusion of the gases. If the contribution of ATH is removed, the diffusion of the gases is then responsible of 42% of the heat evacuation when considering the fast release of gases, or only 21% if assuming slow diffusion. The great difference in efficiency of heat evacuation can be due to the fact that not all the gases have been released at the end of the experiment when assuming slow diffusion. Those data thus demonstrate that, depending on the system, each phenomenon is more or less relevant and the inputs and the sensibility of the prediction to the inputs can be different.

**Figure 19:**
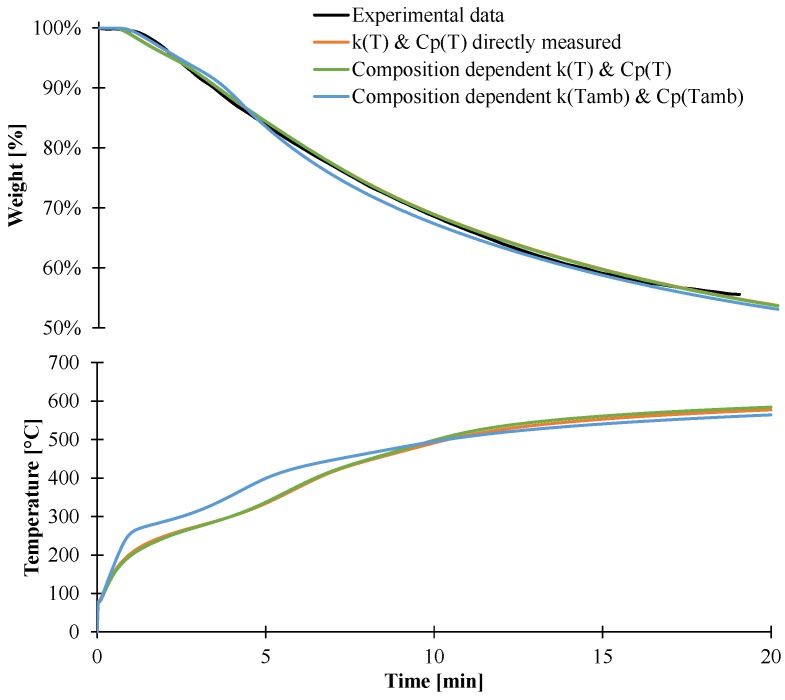
Predicted temperature and weight assuming slow diffusion and different inputs.

**Figure 20:**
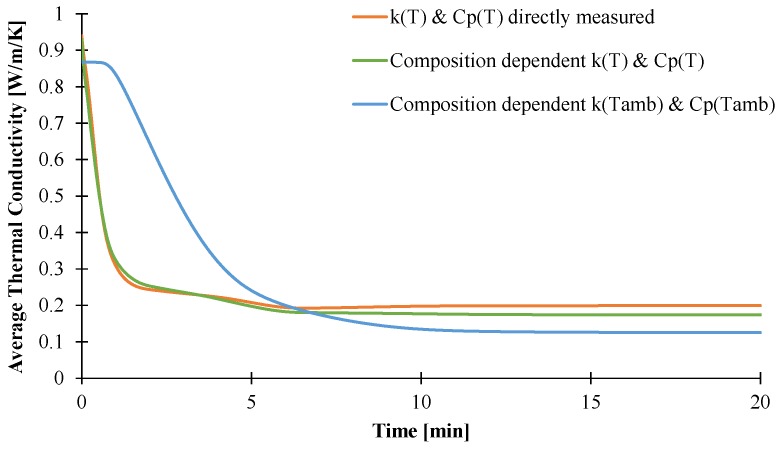
Averaged thermal conductivity assuming slow diffusion and different inputs.

The last objective of this part is to validate the different methods developed in this paper to generate inputs data for the pyrolysis model. Thus, the model was then used considering direct measurement, or thermal conductivity and heat capacity dependent on the composition and temperature, or with values determined at ambient temperature. Figure 19 shows the results obtained considering these approaches. The predicted weight loss assuming slow diffusion and thermal parameters as a function of composition and temperature or just as a function of temperature leads to similar results and is in good agreement with the experimental data. This can be linked with the relative good agreement of the averaged thermal conductivity inside the plaque for both temperature-dependent approaches, as shown in Figure 20. On the contrary, taking into account the composition-dependent thermal parameters at ambient temperature leads to some difference in the weight and temperature profiles. Indeed, the weight after 5 min is slightly under-predicted compared to experimental values even if the temperature is up to 60 °C higher. The temperature at the backside of the plaque is higher due to a higher conductivity inside the plaque during the first 5 min compared to temperature-dependent thermal parameters.

Those results demonstrate that the methods used to determine the thermo-physical properties of materials are of prime interest to be implemented in pyrolysis modeling. As previously stated, determination of a material‘s properties at ambient temperature is an easy task, but is not sufficient enough to predict the behavior of decomposition material well. On the contrary, these results also show that direct measurement is as efficient as “stepped” methods which are time-consuming. As consequence, the direct measurement methods appear as a good compromise to determine the thermo-physical properties of EVA/ATH and potentially flame-retardant materials. 

## 4. Conclusions

Pyrolysis modeling of flame-retardant materials is of prime interest for the scientific community. This approach needs the determination of accurate input data. The objective of this paper was to develop and to compare several methods used for the determination of the thermal properties of materials and to evaluate their impact on pyrolysis models. Special considerations for the characterization of the input have been made as materials undergo multiple-step decomposition. Different characterization tools such as isothermal modulated DSC or dynamical standard DSC (refers as ASTM E1269-11) as well as the Transient Plane Source method have been applied to an EVA/ATH formulation. By going through the measuring/cooling processes, it is possible to evaluate the thermal properties of each individual decomposition species that is obtained at each step of decomposition. It was found that a good agreement between each of the experimental techniques is obtained. Moreover, optical properties of the material at ambient temperature have been performed but additional considerations have to be addressed in order to measure this property as a function of temperature. The heat capacities of the decomposition gases were also assessed thanks to gasification experiments coupled with quantitative FTIR. 

The characterizations of the thermo-physical properties were then validated, as the mass loss and temperature increase of the gasification of this material were well predicted by a pyrolysis model. It can be noted that the characterization of temperature-dependent properties is of great importance and permits higher precision of the models. It was also shown in this study that the diffusion of gases does not significantly influence the temperature profile due to the presence of ATH, which acts as a heat sink. Nonetheless, tuning the mass transfer rate and, thus, the rate of diffusion of the gases inside the material plays a role on the mass loss profiles, and it was shown that the gasification of EVA/ATH can be modeled only by the assumption of slow diffusion of the pyrolyzates. Indeed, the release of each gas was well predicted by low D values. It could be interesting to investigate the impact of this slow diffusion on the internal pressure by substituting the mass transfer sub-model with Darcy’s law. Even if the characterization of permeability or gas viscosity as a function of temperature and decomposition state can be difficult, these simulations can be valuable. 

## Nomenclature

*General parameters/variables*			
*t*	Time	*x*	Depth
*T*	Temperature	*m*	Mass
*N_r_*	Number of decomposition reactions of the material	*N_g_*	Number of gaseous component released by the material
*Optical properties*			
*M*	Black body excitance	*I*	Irradiance
α	Reflectivity	*A*	Absorption coefficient
ε	Emissivity	*T*	Transmittance
λ	Wavelength	*R*	Reflectance
*d*	Thickness		
*Kinetic parameters*			
${\dot{r}}_{r}$	Reaction rate for reaction *r*	*R*	Ideal gas constant
*A_r_*	Pre-exponential factor	*n*	Order of reaction
*E_r_*	Activation energy for reaction r	$\nu_{r}^{i}$	Stoichiometric coefficient of component i in reaction r
*Thermo-physical properties*			
*Cp*	Heat capacity	ρ	Density
*k*	Thermal conductivity	*H_r_*	Enthalpy of reaction *r*
*D*_gas_ *_i_*	Mass trans transfer coefficient of component i		
